# Combined CatWalk Index: an improved method to measure mouse motor function using the automated gait analysis system

**DOI:** 10.1186/s13104-018-3374-x

**Published:** 2018-04-27

**Authors:** Samuel T. Crowley, Kazunori Kataoka, Keiji Itaka

**Affiliations:** 1 Department of Biofunction Research, Institute of Biomaterials and Bioengineering, Tokyo Medical and Dental University (TMDU), 2-10-3 Kanda-Surugadai, Chiyoda-Ku, Tokyo, 113-8510 Japan; 2 Innovation Center of NanoMedicine (iCONM), Kawasaki Institute of Industrial Promotion, 3-25-14 Tonomachi, Kawasaki-Ku, Kawasaki, 210-0821 Japan

**Keywords:** Spinal cord injury, Locomotor function, Mouse, New scoring, CatWalk, Combined Catwalk Index

## Abstract

**Objective:**

Measuring motor function in mice is important for studying models of spinal cord injury (SCI) or other diseases. Several methods exist based on visual observation of mice moving in an open field. Though these methods require very little equipment, observers must be trained, and the possibility of human error or subjectivity cannot be eliminated. The Noldus CatWalk XT Automated Gait Analysis system assesses mouse motor function by taking high-resolution videos of the mice, with specialized software to measure several aspects of the animal’s gait. This instrument reduces the possibility of human error, but it is not always clear what data is important for assessing motor function. This study used data collected during mouse SCI experiments to create a simple mathematical model that combines the data collected by the CatWalk system into a single score, the Combined CatWalk Index or CCI.

**Results:**

The CCI system produces similar results to the Basso Mouse Scale or the CatWalk’s Step Sequence Regularity Index. However, the CCI has a significantly smaller coefficient of variation than either other method. Additionally, CCI scoring shows slightly better correlation with impact force. The CCI system is likely to be a useful tool for SCI research.

**Electronic supplementary material:**

The online version of this article (10.1186/s13104-018-3374-x) contains supplementary material, which is available to authorized users.

## Introduction

Spinal cord injury (SCI) research often relies on animal models. Several methods exist to create animal models of SCI and to assess motor function recovery after injury. Open-field locomotion methods are particularly popular because they require very little equipment. Any flat surface large enough for the animal to freely walk on is usually acceptable. The Basso-Beattie-Bresnahan (BBB) Scale [1] was developed to measure motor function in rats. The Basso Mouse Scale (BMS) [2] was developed later to accommodate for the differences in motor function recovery between mice and rats. The BMS system has been further modified and extended to create other measurement scales, such as the Toyama Mouse Scale (TMS) [3], which is designed to emphasize weight support and reduce the ambiguity seen in the BMS, which can assign the same score to mice with different combinations of stepping frequency, coordination, etc. When properly performed, these methods are reliable and repeatable. However, all of these methods require trained observers. To increase reliability, mice should be observed by at least 2 different observers, and the observers must be blinded to the experiment. Not all laboratories are able to meet these requirements, and the possibility of human error or subjective measurement cannot be ruled out.

More objective methods to assess mouse motor function usually require some form of instrument or other equipment. The Noldus CatWalk XT Automated Gait Analysis System [4, 5] uses an instrument with a glass platform above a high-resolution video camera. Green light is internally reflected through the glass platform. As the mouse walks along the platform, light is reflected down towards the camera wherever the mouse contacts the glass. The intensity of the reflected light is proportional to the pressure placed on the glass. Proprietary software analyzes the videos and produces a large amount of data to describe several aspects of how the animal walks, including speed, timing, coordination, etc. The software produces approximately 25 different measurements for each paw, plus measurements for the mouse overall, for a total of approximately 104 parameters. The large amount of data produced by the CatWalk software is too much to reasonably present everything in a single publication, so a subset of parameters must be chosen. This choice can be arbitrary, and it is difficult to know if the chosen parameters are relevant or adequate for a given experiment. Researchers may be tempted to present any parameter that shows a desired statistically significant result, even if that parameter is not actually relevant to the study.

This study was carried out to produce a method that combines all CatWalk parameters into a single score, the Combined CatWalk Index (CCI), so that the results are easier to compare and report. The data used in this study was collected during a series of experiments utilizing a mouse model of SCI.

## Main text

Several SCI experiments were performed using female C57BL/6J mice (Charles River Japan, Yokohama, Japan), including experiments to test potential therapeutics as well as impact force optimization experiments. The data used in this study was collected from approximately 800 measurements taken from 108 mice.

Mice were anesthetized using an intraperitoneal injection of a mixture of 0.3 mg/kg Medetomidine, 4 mg/kg Midazolam, and 5 mg/kg Butorphanol. Anesthesia was confirmed by pinching the hind paw prior to surgery. The spinal cord was exposed by laminectomy at the 11^th^ thoracic vertebra. Contusion SCI was performed using the Infinite Horizons IH-0400 Impactor [6] (Precision Systems and Instrumentation LLC, Fairfax, VA, USA) using peak impact forces between 40 and 70 kdyne. The animal’s bladders were manually pressed every day to drain the urine and prevent urinary tract infections. After all motor function measurements were completed, mice were anesthetized again and euthanized by cervical dislocation.

Motor function was monitored using the Basso Mouse Scale (BMS) and Noldus CatWalk XT (Noldus Information Technology, Wageningen, The Netherlands) every week for 6 weeks post-injury.

BMS data was collected by observing a mouse in a 30 × 30 × 15 cm plastic cage for 5 min. Notes were taken describing several aspects of the animal’s gait, and BMS scores were calculated according to a flowchart [2].

CatWalk data was collected using a gain of 0.18, with a maximum compliant run time of 12.5 s. When possible, three compliant runs were recorded for each mouse, but poorly performing mice were often unable to produce runs faster than 12.5 s. In these cases, three non-compliant runs were collected. Runs were rejected if the animal turned around during the run. Post-injury data from each mouse was compared to a pre-injury baseline made of three pre-injury measurements to account for naturally occurring variation in motor function.

**Fig. 1:**
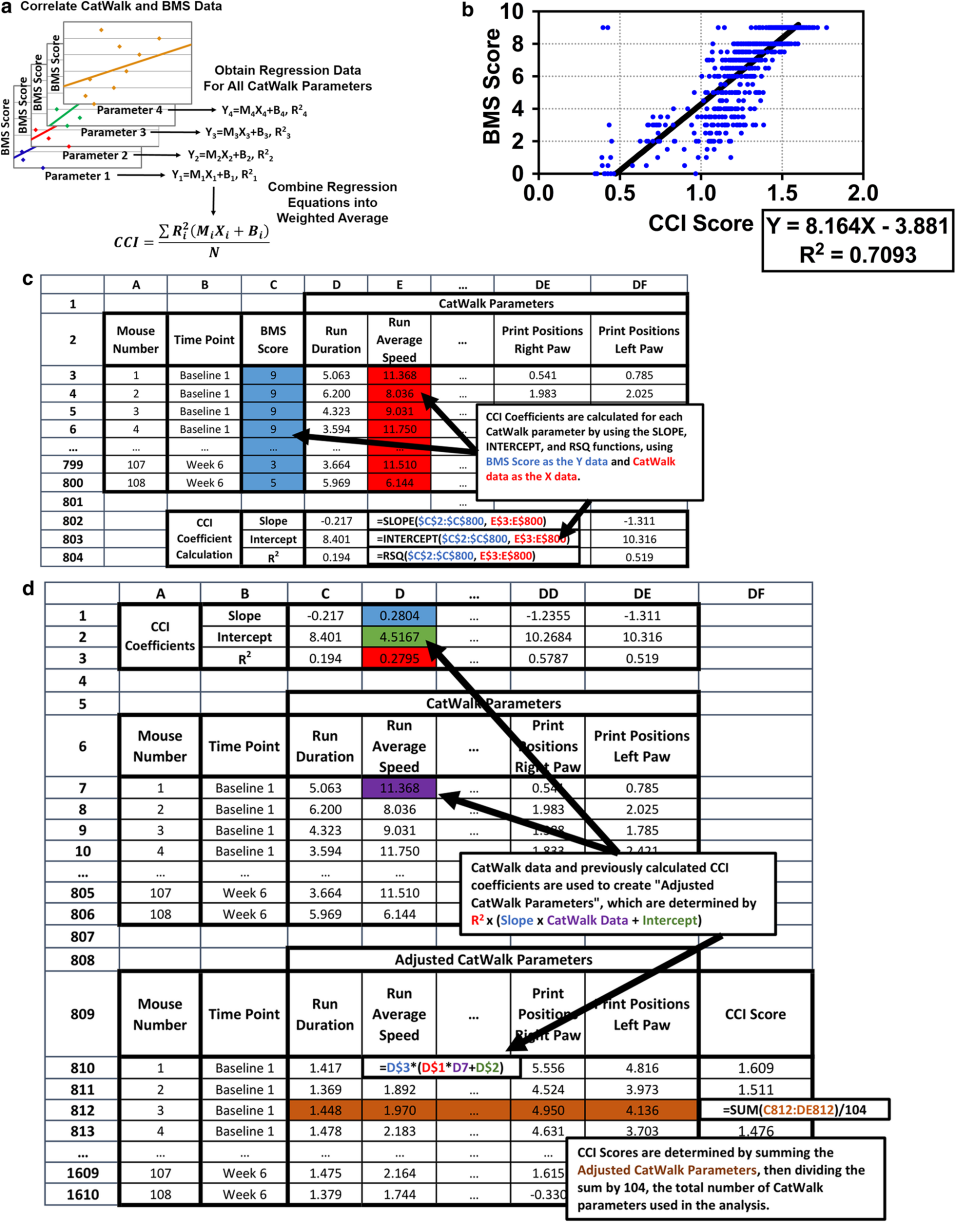
**a** Schematic of how CCI scores are calculated. Data from N CatWalk parameters is correlated with BMS data using linear regression. Each parameter produces a slope (M), Y-Intercept (B), and R^2^, which are listed in Table 1. The CCI score is then determined using a weighted average of each CatWalk parameter using MX + B linear equations multiplied by R^2^ as the weighting factor. **b** Plot of CCI scores against corresponding BMS scores. CCI scores correlate with BMS scores with an R^2^ value of 0.7093, slightly higher than the CatWalk parameter with the highest R^2^ value (Step Sequence Regularity Index, R^2^ = 0.7048). **c** Example of how the CCI coefficients are determined from BMS to CatWalk data using a spreadsheet. Linear regressions are performed for each CatWalk Parameter using the SLOPE, INTERCEPT, and RSQ functions. **d** Example of how CCI scores are calculated from CCI coefficients and CatWalk data. Each CatWalk parameter is multiplied by its CCI coefficients to create an “Adjusted CatWalk Parameter”. The adjusted CatWalk parameters are summed and divided by 104, the number of CatWalk parameters used to create the CCI coefficients

The Combined CatWalk Index (CCI) was developed by correlating 104 CatWalk parameters against observed BMS scores using linear regression, then combining all linear regression equations into a single weighted average (Fig. 1a). The R^2^ values are used as the weighting values, so that parameters with strong correlation have strong weights, while poorly correlating parameters have weak weights. Calculations were performed using both Microsoft Excel 2013 and LibreOffice Calc version 5, both programs produced identical results. Linear regression was performed on each parameter using the built-in SLOPE, INTERCEPT, and RSQ functions, which calculate the slope, Y intercept, and R^2^ values for a linear regression equation (Fig. 1c). Using these formulas is much simpler than preparing a separate scatterplot for each parameter and linear regression. BMS scores were used as the Y-axis data and CatWalk parameters were used as the X-axis data. The CCI coefficients (slope, intercept, and R^2^ values) for each parameter are listed in Table 1, in order from highest to lowest R^2^.

**Table 1:** List of CatWalk parameters and their slope, intercept, and R^2^ coefficients determined by linear regression against BMS data

Rank	CatWalk parameter name	Slope	Intercept	R^2^	Rank	CatWalk parameter name	Slope	Intercept	R^2^
1	StepSequence_RegularityIndex_(%)	0.070	2.486	0.705	53	RF_PrintArea_(cm^2^)_Mean	− 17.88	8.937	0.119
2	PrintPositions_RightPaws_Mean_(cm)	− 1.235	10.268	0.579	54	RH_StepCycle_(s)_Mean	− 1.213	7.970	0.118
3	LH_MaxContactMeanIntensity_Mean	0.222	− 11.437	0.519	55	RF_MaxContactArea_(cm^2^)_Mean	− 21.83	8.790	0.113
4	PrintPositions_LeftPaws_Mean_(cm)	− 1.311	10.316	0.519	56	RH_PrintArea_(cm^2^)_Mean	11.857	6.034	0.109
5	LH_MeanIntensity_Mean	0.213	− 11.455	0.512	57	RH_Swing_(s)_Mean	− 1.300	7.532	0.108
6	RF_DutyCycle_(%)_Mean	− 0.154	17.709	0.497	58	LH_MaxIntensityAt_(%)_Mean	0.071	4.116	0.103
7	RH_MaxContactMeanIntensity_Mean	0.203	− 9.815	0.486	59	LH_StandIndex_Mean	− 0.227	5.899	0.102
8	RH_MeanIntensity_Mean	0.202	− 10.367	0.478	60	RH_SingleStance_(s)_Mean	− 8.141	8.313	0.098
9	RH_MaxIntensity_Mean	0.057	− 0.639	0.474	61	LH_SingleStance_(s)_Mean	− 5.196	8.041	0.094
10	RH_MaxContactMaxIntensity_Mean	0.059	− 0.496	0.471	62	RF_MinIntensity_Mean	0.622	− 27.47	0.093
11	LH_MaxIntensity_Mean	0.059	− 0.802	0.468	63	RH_StandIndex_Mean	− 0.202	6.057	0.088
12	LH_MaxContactMaxIntensity_Mean	0.061	-0.720	0.467	64	LF_StepCycle_(s)_Mean	− 4.048	8.932	0.088
13	LH_SwingSpeed_(cm/s)_Mean	0.096	3.890	0.467	65	RH_MaxIntensityAt_(%)_Mean	0.065	4.427	0.088
14	LH_MeanIntensityOfThe15MostIntensePixels_Mean	0.072	− 0.771	0.465	66	RF_SwingSpeed_(cm/s)_Mean	− 0.065	9.401	0.087
15	RH_MeanIntensityOfThe15MostIntensePixels_Mean	0.069	− 0.416	0.441	67	RF_StepCycle_(s)_Mean	− 4.139	8.980	0.085
16	LF_DutyCycle_(%)_Mean	− 0.153	17.519	0.437	68	RF_PrintWidth_(cm)_Mean	− 3.121	9.417	0.084
17	LF_SingleStance_(s)_Mean	36.619	2.973	0.429	69	LH_StrideLength_(cm)_Mean	0.406	5.620	0.070
18	RH_SwingSpeed_(cm/s)_Mean	0.085	4.312	0.417	70	LF_MaxContactArea_(cm^2^)_Mean	− 17.90	8.405	0.069
19	RF_StrideLength_(cm)_Mean	1.214	2.546	0.393	71	LF_StandIndex_Mean	− 0.431	5.330	0.069
20	LF_StrideLength_(cm)_Mean	1.189	2.638	0.371	72	LF_PrintWidth_(cm)_Mean	− 2.836	9.171	0.068
21	RF_Swing_(s)_Mean	28.297	3.691	0.368	73	LF_PrintArea_(cm^2^)_Mean	− 13.93	8.456	0.065
22	LF_Swing_(s)_Mean	33.472	3.063	0.365	74	RF_BodySpeedVariation_(%)_Mean	− 0.021	8.099	0.062
23	RF_SingleStance_(s)_Mean	36.982	2.867	0.363	75	LF_BodySpeedVariation_(%)_Mean	− 0.017	7.912	0.049
24	LH_PrintLength_(cm)_Mean	7.436	3.049	0.342	76	RF_MaxContactAt_(%)_Mean	− 0.070	10.161	0.038
25	LH_BodySpeed_(cm/s)_Mean	0.270	4.546	0.341	77	RF_PrintLength_(cm)_Mean	− 4.888	10.046	0.038
26	RH_DutyCycle_(%)_Mean	0.092	1.449	0.337	78	BOS_HindPaws_Mean_(cm)	0.504	5.866	0.038
27	LH_DutyCycle_(%)_Mean	0.099	0.907	0.334	79	RF_MeanIntensityOfThe15MostIntensePixels_Mean	− 0.033	10.442	0.034
28	LH_MaxContactArea_(cm^2^)_Mean	34.560	4.844	0.325	80	RF_MaxContactMeanIntensity_Mean	− 0.120	16.638	0.033
29	RH_BodySpeed_(cm/s)_Mean	0.256	4.659	0.304	81	RH_StrideLength_(cm)_Mean	0.258	6.195	0.033
30	Run_Average_Speed_(cm/s)_Mean	0.280	4.517	0.279	82	RF_MaxIntensityAt_(%)_Mean	− 0.060	10.589	0.033
31	LH_Swing_(s)_Mean	− 4.903	8.134	0.277	83	RF_MeanIntensity_Mean	− 0.093	14.731	0.025
32	RH_MaxContactArea_(cm^2^)_Mean	30.206	5.159	0.268	84	LF_SwingSpeed_(cm/s)_Mean	− 0.035	8.215	0.024
33	RF_BodySpeed_(cm/s)_Mean	0.265	4.422	0.253	85	RF_MaxContactMaxIntensity_Mean	− 0.025	10.206	0.023
34	LF_BodySpeed_(cm/s)_Mean	0.261	4.475	0.250	86	LH_Stand_(s)_Mean	− 1.254	7.615	0.021
35	RH_MinIntensity_Mean	− 0.486	34.320	0.244	87	RF_MaxIntensity_Mean	− 0.021	9.839	0.020
36	RF_InitialDualStance_(s)_Mean	− 9.510	8.244	0.236	88	LH_BodySpeedVariation_(%)_Mean	− 0.012	7.769	0.017
37	LF_TerminalDualStance_(s)_Mean	− 8.467	8.145	0.224	89	RH_BodySpeedVariation_(%)_Mean	-0.004	7.379	0.016
38	RF_TerminalDualStance_(s)_Mean	− 10.22	8.314	0.219	90	RH_Stand_(s)_Mean	− 1.159	7.598	0.016
39	LH_PrintArea_(cm^2^)_Mean	19.742	5.300	0.218	91	LF_MaxContactMeanIntensity_Mean	− 0.070	12.532	0.012
40	RH_MaxContactAt_(%)_Mean	0.087	3.924	0.212	92	LF_MinIntensity_Mean	0.208	− 4.589	0.010
41	LF_InitialDualStance_(s)_Mean	− 10.51	8.312	0.211	93	LF_MeanIntensityOfThe15MostIntensePixels_Mean	− 0.016	8.610	0.008
42	LH_MinIntensity_Mean	− 0.499	35.113	0.207	94	LF_MeanIntensity_Mean	− 0.049	11.034	0.007
43	LH_MaxContactAt_(%)_Mean	0.087	3.900	0.203	95	LH_InitialDualStance_(s)_Mean	− 0.712	7.456	0.004
44	RF_Stand_(s)_Mean	− 5.729	9.090	0.201	96	LF_MaxContactAt_(%)_Mean	− 0.023	7.986	0.004
45	Run_Duration_(s)_Mean	− 0.217	8.401	0.194	97	LF_MaxContactMaxIntensity_Mean	− 0.010	8.206	0.004
46	LF_Stand_(s)_Mean	− 5.481	9.001	0.190	98	Run_Maximum_Variation_(%)_Mean	− 0.006	7.344	0.004
47	LH_StepCycle_(s)_Mean	− 2.866	8.798	0.187	99	RH_TerminalDualStance_(s)_Mean	− 0.840	7.429	0.003
48	RH_PrintLength_(cm)_Mean	4.539	4.648	0.186	100	LF_MaxIntensity_Mean	− 0.007	7.897	0.002
49	BOS_FrontPaws_Mean_(cm)	− 1.572	9.967	0.148	101	LH_TerminalDualStance_(s)_Mean	− 0.357	7.411	0.000
50	LH_PrintWidth_(cm)_Mean	3.110	5.220	0.143	102	LF_PrintLength_(cm)_Mean	− 0.389	7.209	0.000
51	RF_StandIndex_Mean	− 0.582	4.712	0.139	103	LF_MaxIntensityAt_(%)_Mean	− 0.002	7.101	0.000
52	RH_PrintWidth_(cm)_Mean	2.858	5.406	0.130	104	RH_InitialDualStance_(s)_Mean	0.017	7.316	0.000

Parameters are sorted by R^2^ value to place parameters with better correlation at the top

The CCI Score was calculated by combining all equations into a weighted average. “Adjusted CatWalk Parameters” were calculated using the original CatWalk data and CCI coefficients with this equation: R^2^ (Slope × CatWalk Parameter + Intercept) (Fig. 1d). Adjusted CatWalk Parameters were then summed and the sum was divided by 104, the number of CatWalk parameters used in this analysis. Mock ups of the spreadsheets used to calculate the CCI coefficients and CCI scores are shown in Fig. 1c, d, and an example spreadsheet containing the BMS and CatWalk data from impact force optimization experiments is available in Additional file 1.

CCI scores were calculated for every mouse at every time point based on the CatWalk data and the CCI coefficients in Table 1. The CCI scores were plotted against the corresponding BMS measurements and linear regression was used to determine how well the two scores correlated against each other, and an R^2^ value of 0.7093 was obtained (Fig. 1b). This R^2^ value is not perfect, but may be a reflection of the BMS system only being semi-quantitative. For example, if a mouse’s BMS score changes from 1 to 2 (a change from only showing partial ankle movement to full ankle movement without plantar placement), it is not the same as a change from 2 to 3 (the mouse shows plantar paw placement without weight support).

**Fig. 2:**
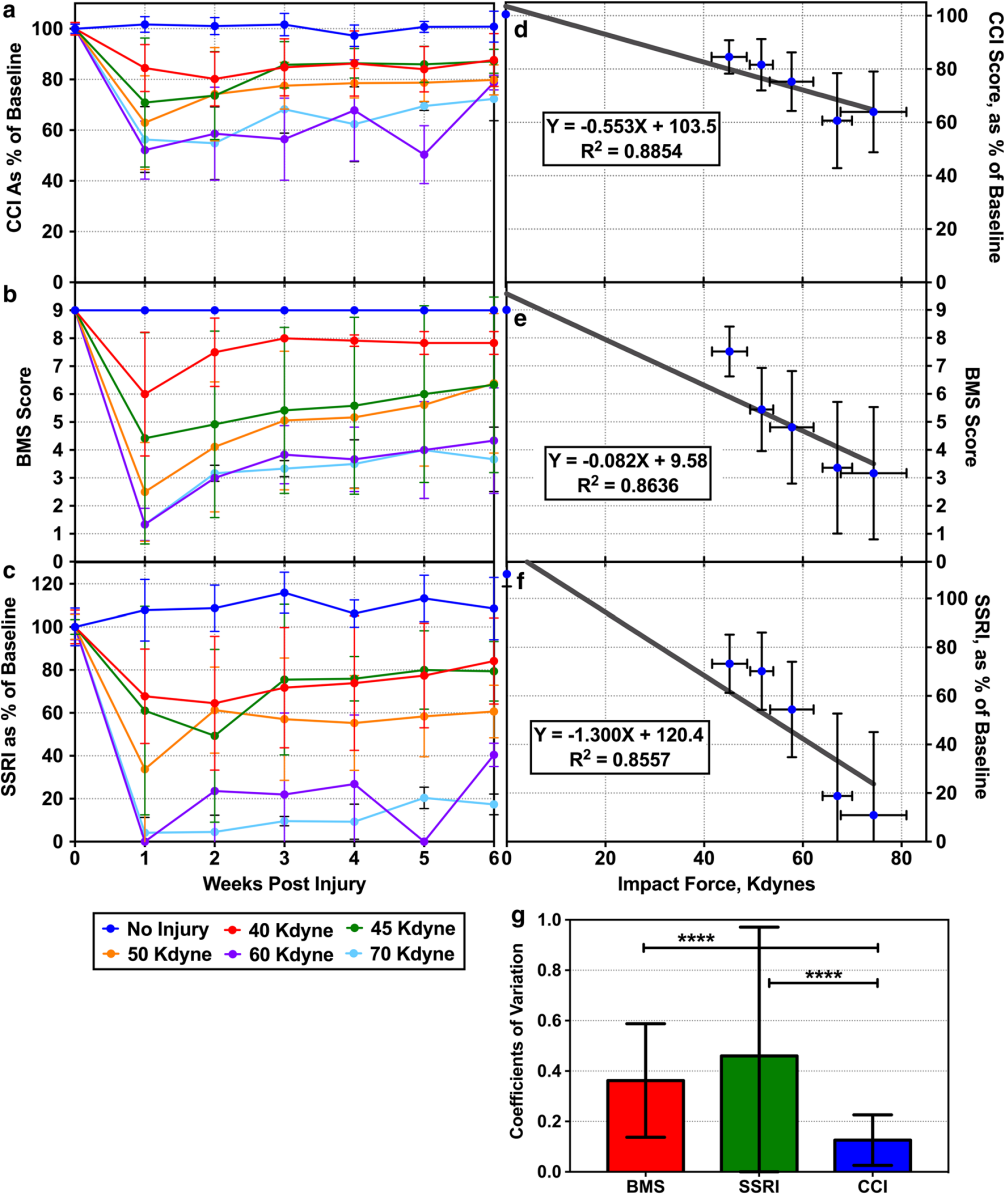
**a**–**c** Comparison of CCI to BMS and SSRI. Mice were given SCI at different impact forces and monitored by BMS and CatWalk methods for 6 weeks post-injury. CCI scores were calculated and plotted in (**a**), while BMS scores are plotted in (**b**). Step Sequence Regularity Index (SSRI) is plotted in (**c**) to represent the CatWalk parameter that mostly closely correlated with BMS score. All three scoring methods show similar patterns of motor function recovery. **d**–**f** Average CCI, BMS, and SSRI scores at each injury level were plotted against impact. CCI produces a slightly better correlation (R^2^ = 0.8854) than BMS (R^2^ = 0.8636) or SSRI (R^2^ = 0.8557). **g** Coefficients of variation (CV) at each time point in plots (**a**–**c**) were determined and averaged. The CCI method has a significantly smaller CV than BMS or SSRI (P < 0.0001 for each, determined by unpaired 2-tailed T Test). All error bars represent sample standard deviation

Table 1 shows that the CatWalk parameter that most closely correlates with BMS scores is the Step Sequence Regularity Index (SSRI), with an R^2^ of 0.7048, slightly lower than the R^2^ value for the correlation between BMS score and CCI score, but this difference is likely insignificant. The SSRI measures coordination by determining if the order of footprints falls into one of six regular patterns. Mice with poor motor function have poor coordination, and do not follow these regular patterns well, producing low SSRI scores. SSRI is often reported in studies that use the CatWalk system [4, 7–11], and the high correlation with BMS scores supports this practice. SSRI was chosen to represent CatWalk data when comparing CCI to CatWalk data in Fig. 2.

Data from the impact force optimization experiments was used to compare CCI scores to BMS scores and SSRI scores. Mean scores and sample standard deviations for each impact force were plotted for every time point (Fig. 2a–c). CCI and SSRI scores are presented as a percent of pre-injury baseline to account for naturally occurring variation between mice. BMS scores are presented directly, because all non-injured mice have a BMS score of 9, so there is no pre-injury variation. All three methods showed similar trends, with higher impact forces producing lower scores. All three methods show fairly large standard deviations, demonstrating the difficulty in producing consistent levels of injury with the contusion SCI model. This is in part due to the difficulty of controlling the Infinite Horizons impactor’s peak impact force. Actual impact forces were usually higher than the desired impact force, with substantial variation.

Average scores for each impact force were estimated by calculating the mean score across weeks 1–6 and were plotted against impact force (Fig. 2d–f). Linear regression was used to determine how well each method correlated with impact force. The CCI scores had a slightly higher R^2^ value (0.8854) than BMS (0.8636) or SSRI (0.8557). This indicates that the CCI score correlates well with the peak impact force in this contusion SCI model.

Coefficients of variation (CV) at each impact force and time point were determined by dividing the sample standard deviation by the mean. Each method’s CVs were averaged and compared using an unpaired, two-tailed T Test (Fig. 2g). The CCI method showed significantly smaller CV than either the BMS method or the SSRI method (P < 0.0001). This implies that the CCI method may be more precise than either other method.

One advantage of the human-observation based BMS method is that it produces a single score that can be easily be compared between mice, but suffers from the potential for human error and the requirement for training. The CatWalk system has the advantage of greater objectivity, but the large number of measurements can complicate several things, such as choosing parameters for publication or making comparisons between mice. For example, if one of set of mice has better coordination, but another set of mice has higher speed, which set of mice has better overall motor function? The Combined CatWalk Index appears to combine the advantages of both system by creating a single number based on objectively determined data. In addition, the CCI scores have slightly better correlation with BMS scores than any individual CatWalk parameter (Fig. 1b, Table 1), slightly better correlation with impact force than BMS scores or SSRI scores (Fig. 2d–f), and significantly smaller coefficients of variation than BMS scores or SSRI scores (Fig. 2g).

Although the CCI method requires a specialized instrument, the CatWalk system is fairly simple, and users can be quickly trained to measure mice. The BBB, BMS, and TMS systems require more extensive training, and steps must be taken to remove human bias or interrater variability. Additionally, the CCI method could potentially be modified and applied to any disease model that can be studied using the CatWalk system, such as chronic pain [12], arthritis, or vestibular disease [11]. The main requirement is to have some semi-quantitative method to rank mice so that correlation between rank and CatWalk parameters can be determined.

## Limitations

The CCI was created by using linear regression of 104 CatWalk parameters against observed BMS scores. The BMS scores were collected by an untrained observer, so the quality of the BMS data might not be optimal, but the decent correlation between BMS score and impact force in Fig. 2e suggests that they are probably sufficient for this analysis. A more significant limitation may be the linear regression model. More sophisticated multivariate regression models exist, but can be more difficult to use with large data sets. For example, the multiple regression method built into Microsoft Excel 2013 can only accept up to 16 variables. The method presented here is a simple extension of linear regression that can be understood and used by almost any researcher using common, or even open source, software.

Finally, good SCI scoring systems should also correlate with spinal cord damage. Systematic histological studies were not performed to verify a link between CCI score and tissue damage, but the close agreement between CCI and the more thoroughly studied BMS and CatWalk systems suggests that CCI is likely to predict spinal cord tissue damage.

## Additional file

**Additional file 1.** Example spreadsheet with CCI coefficients. An annotated example spreadsheet containing CatWalk and BMS data from impact force optimization experiments. This spreadsheet shows how to calculate CCI coefficients and scores.

## Abbreviations

BBB: Basso-Beattie-Bresnahan Rat Scale
BMS: Basso Mouse Scale
CCI: Combined CatWalk Index
CV: coefficient of variation
SCI: spinal cord injury
SSRI: Step Sequence Regularity Index
TMS: Toyama Mouse Scale
